# Supplementary material for: Sugar-mediated regulation of a c-di-GMP phosphodiesterase in Vibrio cholerae
Source: Nat Commun. 2019 Nov 25;10:5358. doi: 10.1038/s41467-019-13353-5 (PMC6877527; doi:10.1038/s41467-019-13353-5)
Supplement: Supplementary file 1 — Supplementary Information [file 41467_2019_13353_MOESM1_ESM.pdf]

Supplementary Information

Sugar-mediated regulation of a c-di-GMP phosphodiesterase in *Vibrio cholerae*

Kyoo Heo[1], Young-Ha Park[1], Kyung-Ah Lee[2,3], Joonwon Kim[4], Hyeong-In Ham[1], Byung-Gee Kim[4,5], Won-Jae Lee[2,3], Yeong-Jae Seok[1*]

[1]School of Biological Sciences and Institute of Microbiology, Seoul National University, Seoul 08826, Republic of Korea

[2]School of biological Sciences and National Creative Research Initiative Center for Hologenomics, Seoul National University, Seoul 08826, Republic of Korea

[3]Institute of Molecular Biology and Genetics, Seoul National University, Seoul 08826, Republic of Korea

[4]School of Chemical and Biological Engineering and Institute of Molecular Biology and Genetics, Seoul National University, Seoul 08826, Republic of Korea

[5]Institute of Engineering Research, Seoul National University, Seoul 08826, Republic of Korea

[*]To whom correspondence should be addressed:

Tel: 82-2-880-4414; Fax: 82-2-888-4911; E-mail: yjseok@snu.ac.kr

Supplementary Table 1. Bacterial strains and plasmids used in this study

Strain or Plasmid	Genotype or phenotype	Source
**Strains**		
Vibrio cholerae		
N16961	Wildtype, Clinical isolate	
N16961 Δ*pdeS*	N16961 with Δ*pdeS*	This study
N16961 Δ*crr*	N16961 with Δ*crr*	This study
N16961 *crr*(H91A)	N16961 in which the chromosomal $EIIA^{Glc}$ was substituted with dephosphomimetic form (H91A) of $EIIA^{Glc}$	This study
N16961 Δ*pdeS crr*(H91A)	N16961 with Δ*vc1710* in which the chromosomal $EIIA^{Glc}$ was substituted with dephosphomimetic form (H91A) of $EIIA^{Glc}$	This study
N16961 *vc1710::3x FLAG*	N16961 in which the chromosomal VC1710 was tagged with 3x FLAG at its c-terminus	This study
N16961 Δ*vpsA*	N16961 with Δ*vpsA*	This study
N16961 Δ*vpsA pdeS*	N16961 with Δ*vpsA pdeS*	This study
Escherichia coli		
ER2566	F^- I^- *fhuA2* [lon] *ompT lacZ*::*T7* gene 1 *gal sulA11* Δ(*mcrC*-mrr)114::IS10 R(*mcr*-73::miniTn10-TetS)2 R(*zgb*-210::Tn10)(TetS) *endA1* [*dcm*]	New England Biolabs
SM10 λ*pir*	*thi-1 thr leu tonA lacY supE recA*::RP4-2-Tc::Mu λ*pir*, OriT of RP4, Km^r; conjugational donor	(Miller *et al*.,1988)
**Plasmids**		
pDM4	Suicide vector for homologous recombination into *V. cholerae* chromosome, OriR6K, Cm^r	(Milton *et al*., 1996)
pETDuet-1		Novagen
pJK1113	pBAD24 with *oriT* of RP4 and *nptI*, P_{BAD}; Km^r , Amp^r	(Lim *et al*., 2014)
pBAD-MycHisA		Invitrogen
pDM4-*pdeS*	pDM4-based suicide vector for deletion of *pdeS*, Cm^r	This Study
pDM4-*crr*	pDM4-based suicide vector for deletion of *crr*, Cm^r	This Study
pET-$HisEIIA^{Glc}$	pETDuet-1-based expression vector for His-$EIIA^{Glc}$, Amp^r	This Study
pET-$EIIA^{Glc}$	pETDuet-1-based expression vector for $EIIA^{Glc}$, Amp^r	This Study
pET-PdeS	pETDuet-1-based expression vector for PdeS, Amp^r	This Study
pET-VC1710::3x FLAG	pETDuet-1-based expression vector for 3x FLAG tagged VC1710, Amp^r	This Study
pET-VCA1085	pETDuet-1-based expression vector for VCA1085 (VcFapA), Amp^r	This Study
pBAD-PdeS	pBAD-MycHisA-based expression vector for PdeS, Amp^r	This Study
pBAD-PdeS(E450A)	pBAD-MycHisA-based expression vector for PdeS(E450A), Amp^r	This Study
pJK-$EIIA^{Glc}$	pJK1113-based expression vector for $EIIA^{Glc,}$ Amp^r, Km^r	This Study
pJK-$EIIA^{Glc}$(H91A)	pJK1113-based expression vector for $EIIA^{Glc}$(H91A), Amp^r, Km^r	This Study
pJK-P_{lac}::GFP	pJK1113-based expression vector for GFP driven by the *lac* promoter, Amp^r, Km^r	This Study

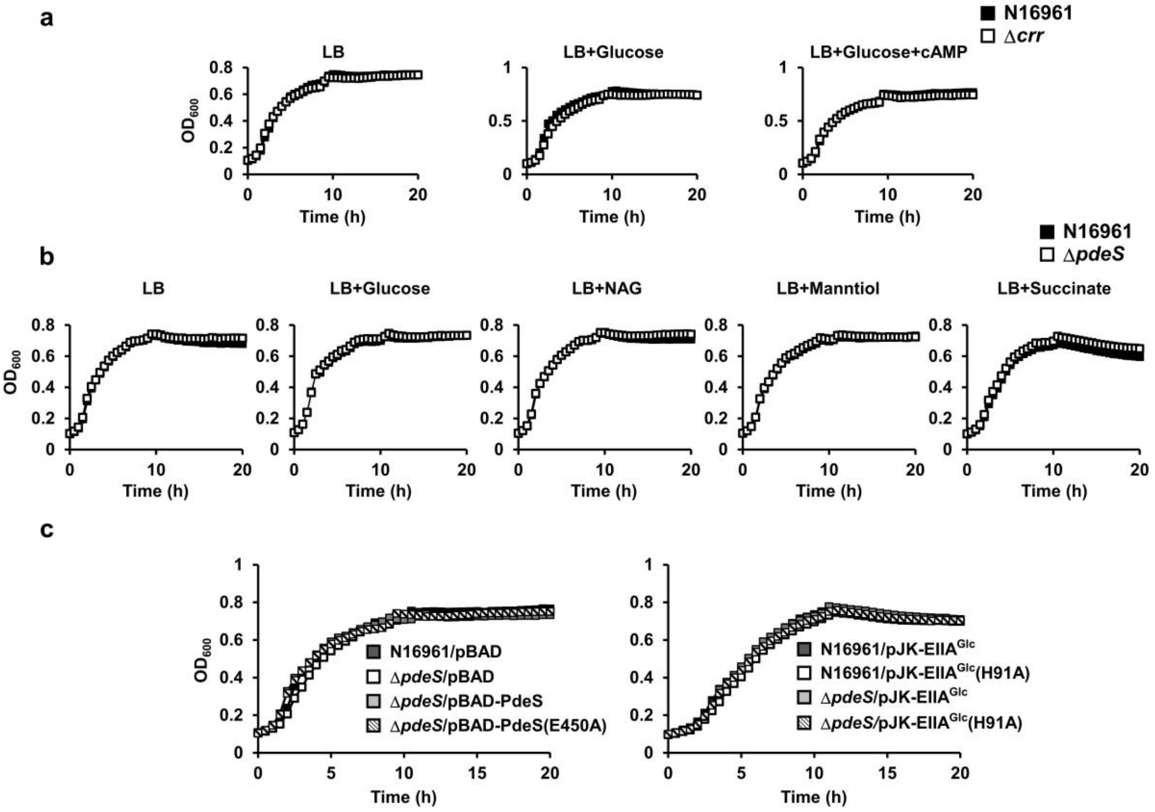

Supplementary Fig. 1 Comparison of growth curves of the *V. cholerae* strains used in this study.

Growth curves of Δ*crr* mutant **(a)** and Δ*pdeS* mutant cells **(b)** were compared with the wild-type *V. cholerae* N16961 strain in buffered LB medium supplemented with the indicated compounds (carbon sources to 0.1%, cAMP to 5 mM). **c,** Growth curves of the indicated *V. cholarae* strains were measured in buffered LB medium supplemented with 0.1% arabinose. The bacterial growth was recorded in triplicates by measuring the absorbance at 600 nm at 37 ˚C in a multimode microplate reader (TECAN).

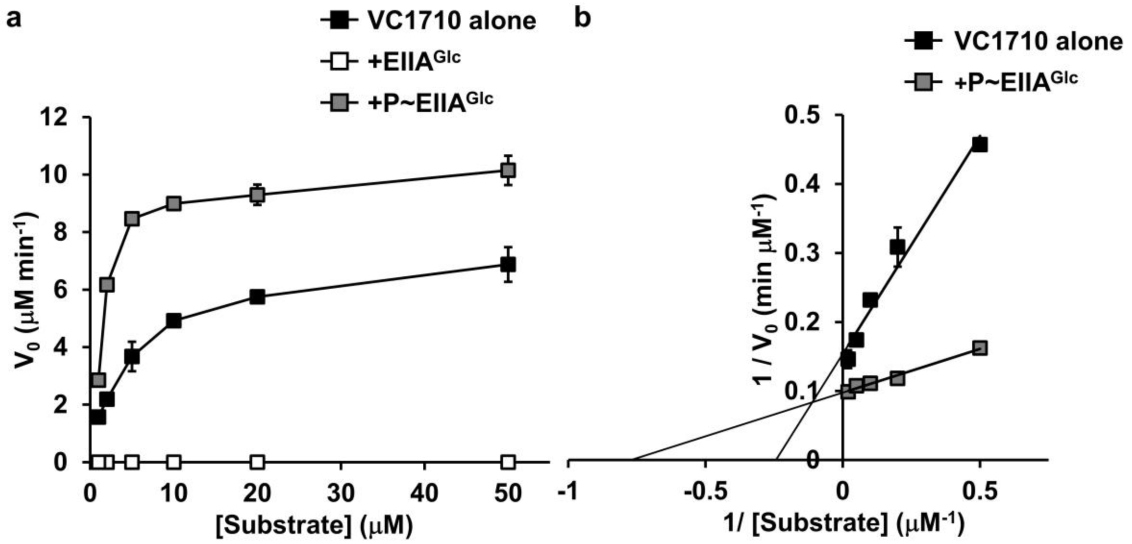

	K_m (μM)	V_{max} (μM min^{-1})
VC1710 alone	4.58±0.12	6.88±0.60
+ P~$EIIA^{Glc}$	1.30±0.08	10.15±0.51

Supplementary Fig. 2 Kinetic properties of c-di-GMP hydrolysis by VC1710.

The Michaelis-Menten constant (K_m) and the maximum initial velocity (V_{max}) were determined for the VC1710-catalyzed hydrolysis of c-di-GMP. **a,** The initial velocity (V_0) of c-di-GMP hydrolysis was determined as described in Methods with 4.1 μM of VC1710 and was plotted as a function of c-di-GMP concentration (black square). The effect of $EIIA^{Glc}$ on the enzyme kinetics of VC1710 was examined by adding approximately a 4 times molar excess of dephosphorylated (white square) or phosphorylated (gray square) $EIIA^{Glc}$ to the reaction mixture. $EIIA^{Glc}$ was phosphorylated by adding 2 mM PEP and 0.4 μM of EI and HPr. **b,** Lineweaver-Burk plots of the data in panel **a**.

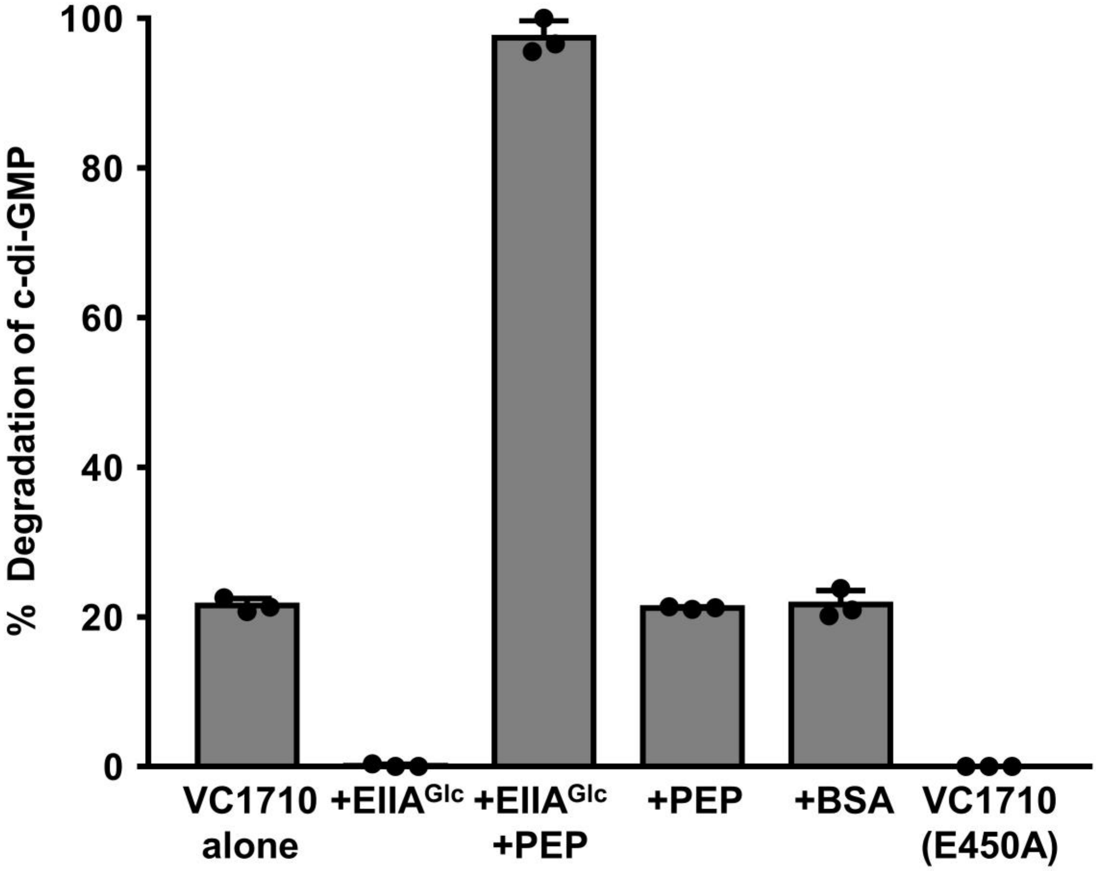

Supplementary Fig. 3 Specific regulation of the c-di-GMP hydrolysis activity of VC1710 by EIIA^{Glc}.

VC1710 (1.58 μM) was mixed with 0.4 μM of EI and HPr, and its c-di-GMP hydrolysis activity was measured in the presence of different combinations of 2 mM PEP, 7.4 μM EIIA^{Glc}, and 7.4 μM BSA as indicated. The active site mutant (E450A) of VC1710 was used as a negative control of the PDE activity.

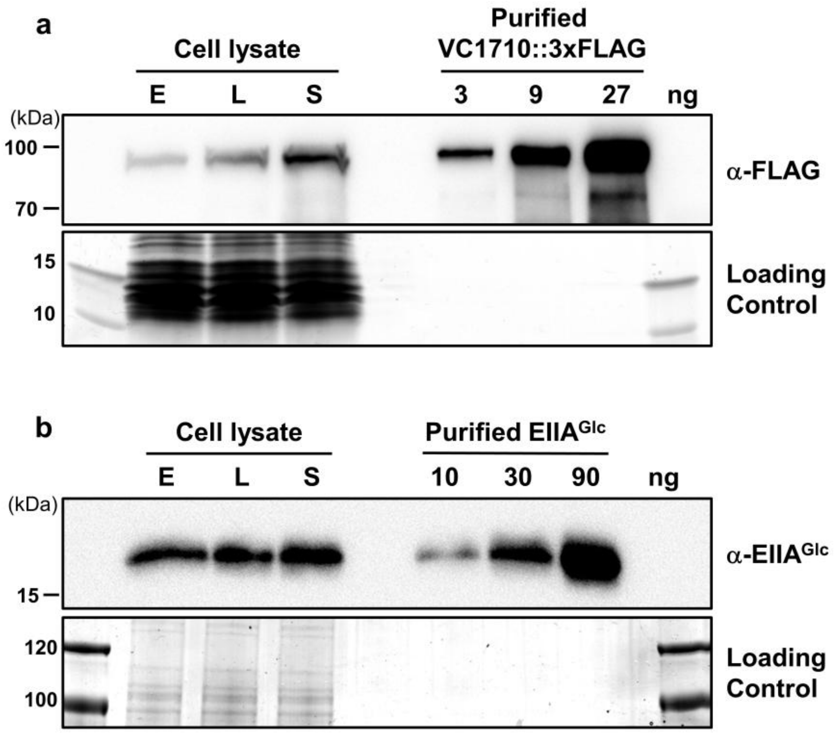

c

# of proteins	VC1710	EIIA^{Glc}
Early Exp.	29~40	17,502~21,668
Late Exp.	68~87	19,792~24,272
Stn.	126~153	22,784~30,803

Supplementary Fig. 4 Quantification of EIIA^{Glc} and VC1710 in *Vibrio cholerae*.

A recombinant *V. cholerae* strain in which the chromosomal VC1710 was tagged with 3xFLAG at its C-terminus was cultured in LB medium and harvested at early exponential phase (E, OD_{600} 0.38~0.48), late exponential phase (L, OD_{600} 1.0~1.15), and stationary phase (S, OD_{600} 1.5~1.6). Cell lysates, along with purified VC1710::3xFLAG (3, 9, 27 ng) **(a)** or EIIA^{Glc} (10, 30, 90 ng) **(b)**, were electrophoresed and subjected to western blot using either α-FLAG monoclonal antibody **(a)** or α-EIIA^{Glc} anti-serum **(b)**. The amount of each protein was determined from the band intensity using ImageJ software. **c,** The cellular copy numbers of VC1710 and EIIA^{Glc} proteins were calculated assuming one *V. cholerae* cell volume of 1×10^{-15} L[4].

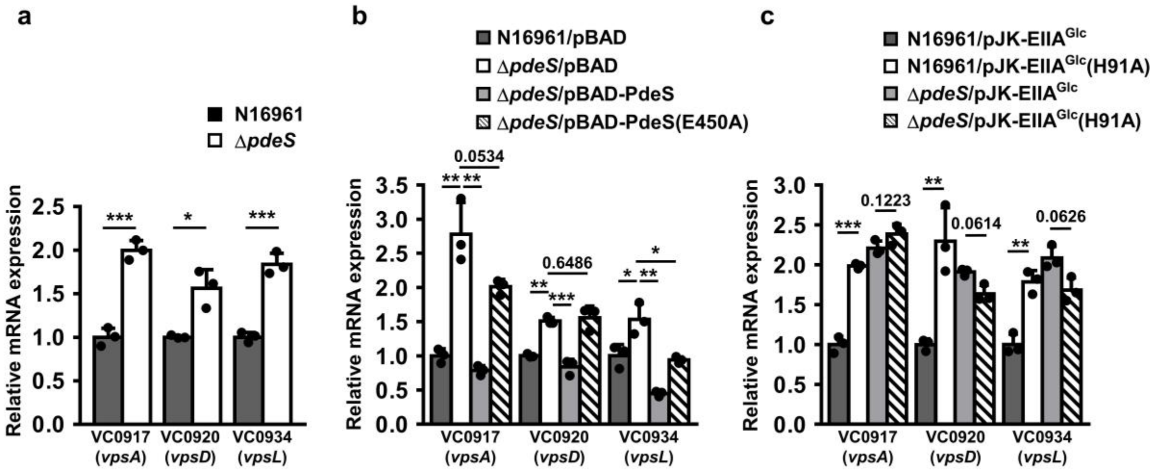

Supplementary Fig. 5 Effects of PdeS and EIIA^{Glc} on expression of the *Vibrio* polysaccharide synthesis (*vps*) cluster genes.

Total mRNA was isolated from the indicated strains grown in LB medium **(a)**, or LB medium containing 0.1% arabinose to induce the expression of PdeS **(b)** or EIIA^{Glc} **(c)**, respectively. The transcription level of genes in the *vps* cluster was measured by qRT-PCR using gene-specific primers for *vpsA*, *vpsD*, and *vpsL*. The expression values were normalized to the expression level of the *rpoB* housekeeping gene. Statistical significance was assessed using Student's *t*-test (*p* values greater than 0.05 were presented in the figure, **p* value <0.05, ***p* value <0.01, ****p* value <0.005). Shown are the means and SD (n=3, independent measurements).

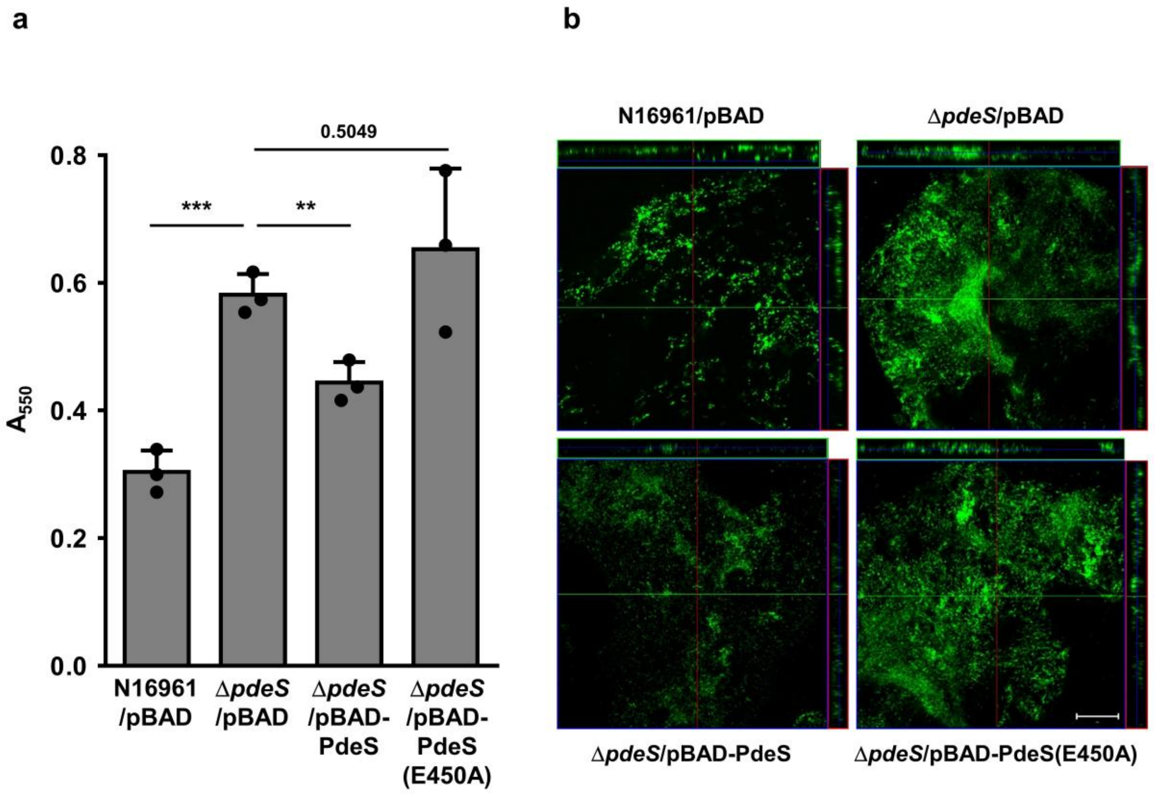

Supplementary Fig. 6 PdeS regulates biofilm formation through its c-di-GMP phosphodiesterase activity.

a, The biofilm-forming activity of wild-type and *pdeS* mutant *V. cholerae* strains harboring pBAD (control vector), or a pBAD-based expression vector for either wild-type PdeS or its active site mutant PdeS(E450A) was measured in LB medium containing 0.1% arabinose. Statistical significance was assessed using Student's *t*-test (*p* values greater than 0.05 were presented in the figure, ***p* value <0.01, ****p* value <0.005). Shown are the means and SD (n=3, independent measurements). **b,** Biofilm formation was visualized using confocal laser scanning microscopy. Scale bar: 50 μm

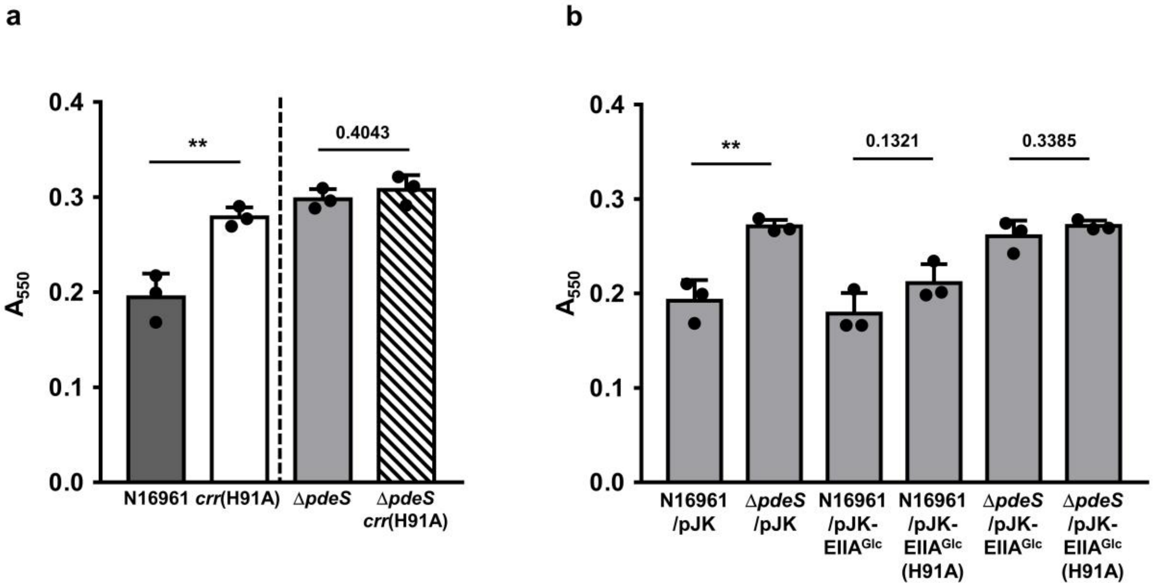

Supplementary Fig. 7 Regulation of biofilm formation by EIIA^{Glc} is dependent on PdeS.

a, The biofilm formation by the wild-type *V. cholerae* N16961 strain and its derivatives carrying *crr*(H91A) and/or *pdeS* mutations was quantified. **b,** The biofilm-forming activity of the wild-type *V. cholerae* and the Δ*pdeS* mutant harboring a pBAD-based expression vector for either wild-type EIIA^{Glc} or dephosphomimetic mutant EIIA^{Glc}(H91A) was determined in the absence of an inducer.

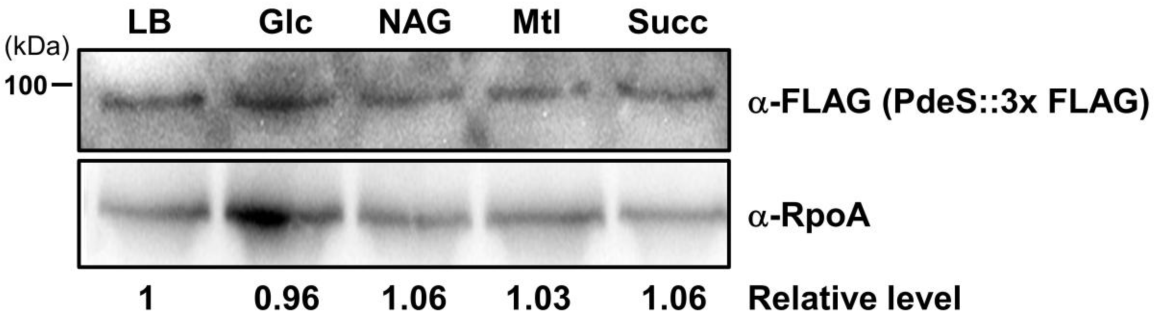

Supplementary Fig. 8 Effect of carbon sources on PdeS expression.

The level of PdeS protein was determined in a *V. cholerae* strain in which the chromosomal PdeS was tagged with 3xFLAG at its C-terminus grown in LB medium or LB medium supplemented with the indicated carbon sources at OD_{600} ~1.0. Cell lysates were analyzed by western blot using α-FLAG monoclonal antibody. The level of PdeS was determined by measuring the band intensity using ImageJ software followed by normalization with that of RNA polymerase α subunit (RpoA). The levels of PdeS were expressed relative to that of PdeS in LB medium, which was set to 1. Glc, glucose; NAG, N-acetylglucosamine; Mtl, mannitol; Succ, succinate.

Supplementary references

1. Miller, V.L. & Mekalanos, J.J. A novel suicide vector and its use in construction of insertion mutations: osmoregulation of outer membrane proteins and virulence determinants in *Vibrio cholerae* requires *toxR*. *J Bacteriol* **170**, 2575-2583 (1988).
2. Milton, D.L., O'Toole, R., Hörstedt, P. & Wolf-Watz, H. Flagellin A is essential for the virulence of *Vibrio anguillarum*. *J Bacteriol* **178**, 1310-1319 (1996).
3. Lim, J.G., Bang, Y.J. & Choi, S.H. Characterization of the *Vibrio vulnificus* 1-Cys peroxiredoxin Prx3 and regulation of its expression by the Fe-S cluster regulator IscR in response to oxidative stress and iron starvation. *J Biol Chem* **289**, 36263-36274 (2014).
4. Park, Y.H., Lee, C.R., Choe, M. & Seok, Y.J. HPr antagonizes the anti-σ^{70} activity of Rsd in *Escherichia coli*. *Proc Natl Acad Sci U S A* **110**, 21142-21147 (2013).
